# A Few Observations on the Therapeutic Value of Electricity

**Published:** 1890-12

**Authors:** Ernest Wende

**Affiliations:** Clinical Professor of Dermatology, University of Buffalo; 174 Franklin Street


Buffalo Medical and Surgical Journal

Vol. XXX. DECEMBER, 1890. No. 5.

### Original Communications.

### A FEW OBSERVATIONS ON THE THERAPEUTIC VALUE OF ELECTRICITY.

By ERNEST WENDE, M. D., B. Sc.,
Clinical Professor of Dermatology, University of Buffalo.

Recently a most fruitful field in galvano-therapeutics has been openedup, and not withstanding that the science of electro-therapeutics is largely experimental, the progress in the use of the continuous or constant current has been rapid, improved apparatus, improved and accurate modes of application, effects carefully ascertained and certified, have done away with the vagueness of aim and the uncertainty of result. Well-directed experiments and fresh discoveries have closely followed each other.

It is not my intention in these brief notes to discuss the modern theories of electricity, nor even the fundamental laws which govern it, but simply to cite a few cases which have come under my observation, and which I hope may prove of some interest:

Case I.—W. M., by occupation a thresher; aged forty-nine; living in the town of Bennington, Wyoming county, called at the office July 14th, to consult Dr. Starr concerning a "Film growing on my eye-ball," as he expressed it. On being told that the doctor had gone to Europe, he desired to consult Dr. Abbott; he also being away, the patient returned to the office, when on a careful examination, I found that the so-called film was the ordinary pterygium, situated in the direction of the internal rectus. It had the usual triangular shape, with its apex attached to the corneal edge. Its color was of a pinkish hue. It was rather thick, and a number of distinctly visible blood-vessels coursed through the growth.

After some persuasion the patient finally submitted to electro-puncture, under a ten per cent. solution of cocaine. I first obliterated the blood-vessels, which was followed by numerous punctures of the membrane itself. The insertions were made with an iridio-platinum needle, as the negative electrode, while the ordinary electro-positive sponge electrode was held in the hand by the patient. I next applied a cold water compress with directions to continue the same.

It was July 23d when I first saw the patient after the operation. The eye was much reddened and presented numerous minute spots denuded of conjunctiva. The growth, however, had virtually disappeared. I again saw him August 13th. The eye at this time was still somewhat injected and discolored. The discoloration being due in all probability to extravasated blood, the fluid having become absorbed, while the corpuscles and coloring matter remained behind, yet the globe of the eye presented a smooth appearance with no trace of the pterygium. The sitting lasted about fifteen minutes, and the strength of the current employed varied from four to seven milliampères.

Case II.— E. B., a strong, healthy-looking young fellow; aged twenty-five; consulted Dr. Hoddick of this city, about one year ago, for a supposed deep stricture of the urethra. He gave a history of having had gonorrhea on several occasions, followed by the usual methods of treatment. One year prior to this, while in the city of Rochester, he was suddenly seized with an attack of retention.

He immediately consulted a local physician, who advised and practised electrolysis of the urethra, with a result which proved almost disastrous for the symptoms which ensued became so urgent, the febrile disturbance so marked, that his parents were sent for. Subsequent to the consultation with Dr. Hoddick he again experienced retention, which the doctor succeeded in relieving by the administration of the usual hip-baths, morphine, suppositories, etc. He then left town for a few months, but on his return he was again seized with a similar attack. Once more the doctor was summoned, but on this occasion all antispasmodic and antiphlogistic treatment failed, nor could the accumulation of urine be drawn off by means of the catheter. However, the patient was finally relieved by aspirating the bladder per rectum.

A few days later, at the suggestion of Dr. Hoddick, I was invited to see the case. On instituting an examination we encountered no difficulty in passing a number of sounds, and therefore concluded that the trouble was merely spasmodic.

Some months later the patient called me up at mid-night, saying that he could not find Dr. Hoddick, and that he had just returned from Erie. He walked into my office in a stooped manner and appeared extremely restless. He referred to pain and local uneasiness in the lower part of his abdomen. His expression was anxious, and his desire for relief urgent. He had not been able to void his urine since the previous evening. He, furthermore, stated that the retention was occasioned by an attack of diarrhea.

My first thought was to employ an anesthetic and careful catheterism, when it suddenly occurred to me that the pathological significance of reflex irritation in its bearing on retention might be relieved by electricity. This I found to be true by placing one electrode, the positive, on the perineum, the other, the negative, above the pubis, over the bladder. The current employed was the galvanic, the dosage twenty milliampères, and the length of application five minutes.

Immediately the urethral spasm became supplemented by a copious flow of urine, of a dark color and a strong odor. Immediately the patient experienced a sense of relief. Exclamations of joy were frequently uttered during the process of micturition. It was a grateful mitigation of an urgent desire. It was a gratifying result. Dr. Hoddick has since had an opportunity to confirm the value of this plan of treatment.

Case III.—W. H. S., a well-known gentleman of this city, for many years court stenographer, and at one time a medical student, first consulted me for malignant disease May 25, 1889. The affection in question was an epithelioma, situated on the side of the nose, in close proximity to the eye, in fact involving the neighboring parts of both the upper and lower lids. The following is the history, and the treatment as addressed to that terrible disease, from the time of its first occurrence to the present date, written by the patient himself. I will give it verbatim, as there is but little for me to add :

About twenty years ago there first appeared a small growth in appearance, similar to a wart, upon the left side of the nose, at a point about equidistant from the corner of the eye and the ridge of the nose. In the course of a year or so it would occasionally develop a small scab, and upon its being removed either purposely or accidentally, there would exude a small quantity of serum. It would then heal up and be scarcely visible for some weeks, and even months. The formation of the scab became more frequent, and the size of the growth gradually became larger. About thirteen years ago I consulted the late Dr. Miner of this city, who advised its removal by the knife. He removed it in that manner. It was done at a time when he was in feeble health, and without an assistant. It bled very profusely, and from subsequent results I am satisfied there was not enough of the tissue removed. However, it healed up and gave me no more trouble for a year or so, when it began to develop again, and more rapidly than before. I then consulted Dr. Cronyn, who was my family physician, and he advised its removal by erasion, with a sharp spoon. The effect of that treatment was about the same as that given by Dr. Miner. After continuing this treatment for a couple of years, at intervals of from three to six months, he applied a mercurial plaster, but after a few months it returned as before. He advised me to go to the late Dr. Davidson, who was making skin diseases a specialty. He treated it with mild caustics, healing it with a salicylic acid ointment. He continued this treatment for two years and a half, when he informed me that he could do nothing further for it. It had at that time spread, covering a place about five-eighths of an inch across. I then went to a cancer doctor; gave him a history of the case. He predicted that he could cure it in ten weeks, completely. He agreed to charge me nothing unless he effected a complete cure. He applied his cancer plaster on about thirty different occasions, each treatment covering a period of three or four days, and of the most heathenish torture possible to imagine. At the end of two years and a half, when the disease had spread until it covered about four times the surface that it did when he began, and extending into the canthus, I bolted. During the last year of his treatment I had been unable to use my eye for any business purpose whatever. I decided it was better to die a natural death, if necessary, from the progress of the disease than to be tortured.

I next consulted Dr. Wende, who began his treatment one year ago last May. His first treatment by electrolysis was so successful that in less than a week the inflammation which had been present in my eye, and keeping it nearly closed during the entire time of the other cancer doctor's treatment, had almost entirely disappeared. From that day until this I have been able to attend to my business and use my eye daily, and without annoyance. If I was able to endure the treatment without having cocaine injected into the tissues, causing the eye to swell, the effects of the treatment would not be noticeable to the casual observer from any swollen appearance of the eye. When Dr. Wende began his treatment, there was over one square inch of surface of open sore and as much more highly inflamed. At the present time the entire surface actually treated covers less than one-eighth of an inch in diameter, and in only three different points. W. H. S.

In treating the ulceration I first injected a four per cent. solution of cocaine in the surrounding and underlying induration, and then with the ordinary iridio-platinum needle destroyed the abnormal tissue with numerous negative galvano-punctures. The insertions were made in various directions, frequently one above the other, and often at right angles with each other. The current that was allowed to pass varied from five to fifteen milliampéres. I cannot give the exact number of sittings the patient has had, approximately should say about twenty, given at irregular intervals, varying in duration from fifteen to thirty minutes. The infiltration grew gradually less after each treatment. Although not cured, the agonizing pain ceased, and the incessant nervous agitation and distress suspended. However, should I not succeed in entirely removing this dire infection even in this most promising case, it may be truly said that electrolysis has done more to alleviate the suffering, and more toward affording a most likely means of reaching the cause, than the knife or the caustic. But, with due regard to justice, I must admit that in the majority of cases thus treated, the method is tedious and generally unsatisfactory, when the term cure comes into question, yet always palliative when relief is asked for — however, there are cases of superficial epitheliomatous ulcers of the non-infiltrating variety — surrounded by comparatively little induration, which may be dealt with most successfully with electro-puncture. Then, again, the intelligent use of this agent cannot be too strongly recommended in operations about such an important structure as the eye, where you are anxious only to destroy diseased tissue. The other advantages possessed by this treatment may be said to be that —

1. It delays the growth, even if it does not always cure.
2. It allays pain.
3. The patient is not necessarily confined to his room.

Case IV.—C. H. S., a tough but a weary-looking lad of twenty-four; accustomed to alcohol and other things of saturation; after many months' suffering from an incessant and copious discharge of the urethra, presented himself at the Fitch Provident Dispensary for the treatment of verruca acuminata, the so-called venereal wart.

Of these he had many, situated on the glans penis. They were closely aggregated, club-shaped, pointed, tufted, and of a bright red color. They completely encircled and covered the glans with a corona, made up of numerous, irregular, craggy nodulations and lobulations. They represented on the whole not only morphologically but in reality a true coxcomb. One of them, which had grown more luxuriantly than the rest, having attained the size of a walnut, was supernal and overhung the meatus, rendering the process of urination unsatisfactory and harassing. The water, instead of flowing down-hill, spurted sideways and backwards, soiling his clothing, and producing an odor highly offensive.

This fleshy and vascular excrescence yielded readily to electrolysis. It was destroyed by inserting a sharp-pointed steel needle, at different points, through the base of each individual growth, on a level with the skin. The needle employed was the negative, while the ordinary positive sponge-electrode, after being thoroughly moistened, was placed on the thigh. The number of insertions varied with the size of peduncle or wart in question. As soon as the hypertrophied mass turned pale, it was an indication that the tissue would shrivel, dry up and drop off. The time required to accomplish this will also vary with the size; usually one to two minutes will suffice.

In this instance all the smaller growths disappeared after one sitting, while the largest, which was almost sessile, needed four sittings, at intervals of three days. Two to five milliampères were used. I have employed this method for the past three years, and consider it superior to all others. In very sensitive persons I inject a two per cent. solution of cocaine hypodermically — or I produce the anesthesia by electric-cataphoresis by first applying a rubber bandage turniquet posteriorly to the glands, a piece of absorbent cotton saturated in a six per cent. solution of cocaine is wrapped about the glands and warts — over this a number of coils are made with a very flexible copper wire. This constitutes the anode, while the ordinary sponge negative electrode, well moistened, is held tightly against the thigh. Now the current is allowed to pass for six to ten minutes, after which the warts can be destroyed without pain in the manner previously stated. Finally, the treated parts may be dusted with some astringent or desiccating powder.

This method possesses advantages offered by no other form of treatment. As you are aware, excision often produces profuse hemorrhage, which may be difficult to control. Electrolysis causes no blood-shed. Caustics, such as nitric and chromic acids, may be vulgarly termed nasty, as they are likely to spread, discoloring the parts and often producing scars. Electrolysis is cleanly and localized in its action and produces no scars. The actual cautery is no better than caustics. Ligation is tedious and uncertain. Electrolysis is certain and rapid.

174 Franklin Street.

